# Reviewed article: Santos ME, Ferreira RC, Pinheiro EL, Vieira SSA, Senna MIB. Organizational readiness for change and work process of oral health teams in Primary Health Care: an exploratory study in the initial implementation phase of MonitoraSB, Minas Gerais, Brazil, 2024. Epidemiol Serv Saude. 2025:34;e20240802

**DOI:** 10.1590/S2237-96222025v34e20240802.c

**Published:** 2025-10-20

**Authors:** Carla Cristina Enes

**Affiliations:** 1 Pontifícia Universidade Católica de Campinas, Campinas, SP, Brazil

## First round comments

Prezados autores,

o artigo apresenta contribuições valiosas ao investigar a prontidão organizacional e os processos de trabalho das equipes de Saúde Bucal (eSB) no contexto do programa MonitoraSB. Os revisores destacaram a relevância da pesquisa, especialmente pela possibilidade de subsidiar políticas públicas no Sistema Único de Saúde (SUS). Contudo, são necessários algumas ajustes para fortalecer a clareza, a robustez metodológica e a aplicabilidade prática dos resultados.

Seguem os comentários e sugestões mais relevantes que podem auxiliar no aprimoramento do manuscrito:

1. Objetivos e metodologia: Os objetivos poderiam ser refinados com maior especificidade e acompanhados de hipóteses testáveis. Na seção metodológica, sugere-se maior detalhamento sobre os critérios de inclusão e exclusão, os procedimentos de coleta de dados e a justificativa para os testes estatísticos empregados. A inclusão da aprovação do Comitê de Ética no início da seção metodológica é recomendada para fortalecer a conformidade ética do estudo.

2. Discussão e implicações: A discussão poderia explorar mais profundamente os resultados, relacionando-os ao impacto no planejamento e execução das políticas de saúde bucal. A análise das limitações do estudo e a apresentação de direções para futuras pesquisas são aspectos que devem ser expandidos. Adicionalmente, seria interessante considerar a inclusão de análises mais direcionadas sobre as características que atuaram como barreiras ou facilitadores à prontidão organizacional, para enriquecer as implicações práticas.

3. Aspectos gerais: Revisão ortográfica e gramatical detalhada é essencial para assegurar a profissionalidade do texto. Além disso, o resumo pode ser aprimorado ao integrar menções claras aos métodos e resultados principais, facilitando a compreensão e atraindo maior interesse dos leitores.

Recommendation: Major revision

